# Cryptococcal antigenemia and associated risk factors among ART‐naïve and ART‐experienced HIV‐infected peoples at selected health institutions of Mekelle, Northern Ethiopia

**DOI:** 10.1002/mbo3.746

**Published:** 2018-10-02

**Authors:** Kibra Hailu, Selam Niguse, Kiflom Hagos, Mahmud Abdulkader

**Affiliations:** ^1^ Laboratory Department Ayder Comprehensive Specialized Hospital Mekelle Ethiopia; ^2^ Medical Microbiology and Immunology Unit College of Health Sciences, Mekelle University Mekelle Ethiopia

**Keywords:** ART‐experienced, ART‐naive, cryptococcal antigenemia, HIV infected, Mekelle

## Abstract

Cryptococcal infection is a major cause of opportunistic infection in HIV/AID‐infected peoples. We determined cryptococcal antigenemia and cryptococcal meningitis among antiretroviral therapy (ART) initiated and ART‐naive HIV‐infected peoples. A cross‐sectional study was conducted at selected health facilities in Mekelle, Ethiopia. Blood was collected to determine CD4 and plasma cryptococcal antigen (CrAg). CSF CrAg and CSF culture and urease tests were also done. Socio‐demographic and clinical data were collected using a structured questionnaire and clinical chart review. From the enrolled study participants, 267 study participants had complete data, of which, 137 (51%) were females. From the study participants, 140 (52%) and 127 (48%) were ART experienced and ART naïve, respectively. The prevalence of cryptococcal antigenemia was 9 (3.4%). All the study participants, except one (CD4 = 120 cells/mm^3^), had CD4 count less than 100 cells/mm^3^. From CrAg‐positive peoples, 6 (4.7%) were ART naïve. Five CrAg‐positive peoples had cryptococcal meningitis. Being male, rural residence, and being hospitalized were associated with cryptococcal antigenemia. Cryptococcal infection poses a substantial risk of HIV‐positive peoples. This study provides relevant data for CrAg screening interventions in patients with low CD4 cell counts.

## 1 INTRODUCTION

Cryptococcal infection is a major cause of opportunistic infection in HIV/AIDS‐infected peoples. Cryptococcosis is one of the few infectious diseases that can be detected in asymptomatic peoples (Centers for disease control and Prevention (CDC), 2014; Meya, Rajasingham, Nalintya, Tenforde, & Jarvis, 2015). Asymptomatic cryptococcosis patients are positive for serum/plasma cryptococcal antigen (CrAg). The subclinical infectious state is known to precede clinically apparent disease by weeks to months, which eventually will lead to meningitis and mortality in HIV‐infected people (Centers for disease control and Prevention (CDC), 2014; Meya et al., 2015; Rajasingham, Meya, & Boulware, 2012).

The prevalence of cryptococcosis is high in Africa, especially in sub‐Saharan Africa, where it is found up to 28% of HIV‐infected patients with clinical signs of meningitis (Ngouana et al., 2015). In Africa, cryptococcal‐related infections place a high burden on healthcare resources. It causes a severe and often fatal meningoencephalitis in people living with HIV/AIDS, accounting for 42% to 71% of neuromeningeal cryptococcosis‐related deaths in sub‐Saharan Africa (Assogoba et al., 2015).

Testing for cryptococcal antigenemia is effective at reducing morbidity and mortality because patients who receive early antifungal treatment have better outcomes than those who receive delayed treatment (Kaplan et al., 2015; World Health Organization, 2011).

CrAg screening programs have begun in a number of countries with high burden of disease (Kaplan et al., 2015). However, in Ethiopia, a few ART clinics have started CrAg screening procedures. Generating evidence through research will support the existing prevention and control programs (Beyene, Woldeamanuel, Asrat, Ayana, & Boulware, 2013; Reepalu et al., 2015). In Ethiopia, the mortality rate due to cryptococcal meningitis is high (Berhe, Melkamu, & Amare, 2012). High prevalence of cryptococcal antigenemia has also been reported in ART sites in Addis Ababa and Adama (Alemu et al., 2013; Beyene et al., 2013, 2017 ). In contrast to these studies, one study has reported a low prevalence of cryptococcal infection in Adama (Reepalu et al., 2015). The existing studies in Ethiopia have shown a varying prevalence of cryptococcal antigenemia. Nevertheless, further assessment in different geographical regions is needed to address associated factor differences. The previous studies were also unable to assess the magnitude of cryptococcal meningitis. Therefore, this study is aimed at determining the cryptococcal antigenemia and associated factors among ART naïve and ART‐experienced HIV‐infected peoples.

## 2 METHODS

### 2.1 Study setting, population, and design

A cross‐sectional study was conducted at Mekelle, Ethiopia, from September 2016 to January 2017. Mekelle is situated 783 km north of the capital of Ethiopia, Addis Ababa. In the city, there are governmental and private health facilities providing HIV diagnostic and management clinics. This study was done among peoples who get ART‐related healthcare service (both outpatients and inpatients during the study period) from Mekelle Hospital, Ayder Comprehensive Specialized Hospital, and Mekelle Health Center. All HIV‐positive individuals aged 18 years and above with CD4 count less than or equal to 250cells/mm^3^ were eligible for this study. Peoples who had been diagnosed with a cryptococcal infection in the previous 2 years, those who were taking antifungal treatments, and severely ill patients that did not give consent were excluded from the study. A total of 280 study participants were included, considering 84% power to detect a 10% difference, with equal group sizes and two‐sided *p* = 0.05 and prevalence (P) from Adama (Sawadogo et al., 2016).

### 2.2 Data collection

A structured questionnaire was prepared to collect socio‐demographic data (age, gender, and residence) and clinical data (a headache, fever, nausea, cough, vomiting, visual changes and neck stiffness). History record cards were also reviewed for length of ART stay.

### 2.3 Sample collection, handling, and transport

Blood samples (3 ml) were collected by vein puncture in EDTA test tubes from each participant by an experienced laboratory professional. Specimens were transported at room temperature to Ayder Comprehensive Specialized Hospital ART laboratory for analysis. For study participants who had a positive plasma CrAg test, and who showed signs and symptoms of cryptococcal meningitis, 2 ml of cerebrospinal fluid (CSF) was collected following a lumbar puncture (LP) using a 21 gauge LP needle with a sterile test tube. LP was done by an experienced physician at the site of patient admission. CSF was immediately transported to Ayder Comprehensive Specialized Hospital Microbiology laboratory for analysis.

### 2.4 Ethics approval and consent to participate

The study protocol was evaluated and approved by the Research Ethics Review Committee of the College of Health Sciences, Mekelle University, and ethical clearance was obtained. Official support letters were obtained from Mekelle University and Tigray Regional Health Bureau. Moreover, prior to conducting the study, the purpose and objective of the study were described to the participants and a written informed consent was obtained. Laboratory examinations with a direct benefit in the health of the study participants were informed to physicians. The consent involves permission to disseminate the findings of the study through the scientific workshop and publish in reputable journals.

### 2.5 Laboratory testing

All laboratory tests for plasma CrAg test, CD4 testing, CSF CrAg test, and culture test were done at the time of data collection.

### 2.6 CD4 count

CD4 count was determined using FACSCount Immunecytometry analyzer (BD Biosciences, San Jose, USA).

### 2.7 Rapid CrAg lateral flow assay (LFA) test

Plasma and CSF samples were used to detect cryptococcal antigenemia and cryptococcal meningitis, respectively. Cryptococcal Antigen Lateral Flow test (ImmunoMycologics, Inc., Oklahoma, USA) was performed, and results were observed within 10 min and interpreted as per the manufacturer's instruction.

### 2.8 Culture for definitive diagnosis of cryptococcal meningitis

CSF was centrifuged at about 250 g for 10 min. The sediment was inoculated to Sabouraud Dextrose agar on two sterile petri dishes and was incubated at room temperature and at 37°C for up to 14 days. A moist white‐cream colored mucoid colonies appeared 3–4 days of incubation. For positive cultures, urease test was carried out on urea agar in a sterile test tube. A colony from the culture was inoculated and incubated at 37°C for 3–5 hr.

### 2.9 Data quality assurance

Reagents were checked for proper functioning using provided control reagents. CSF was collected aseptically to prevent contamination using 70% alcohol and 2% tincture of iodine. Sterility of media was checked by incubating random samples of prepared culture media at room temperature and at 37°C for 14 days.

### 2.10 Statistical analysis

The data were entered into the EPI Info version 7 (CDC, USA) every day. The data were imported from EPI Info and analyzed using Statistical Package for Social Sciences (SPSS) software version 22.0 (IBM, USA). Descriptive statistics were computed, and data were presented using figures and tables. Association between different variables with outcome was analyzed using Fisher's exact test. P‐values less than 0.05 were considered as statistically significant.

## 3 RESULTS

### 3.1 Socio‐demographic and clinical characteristics of study participants

**Figure 1:**
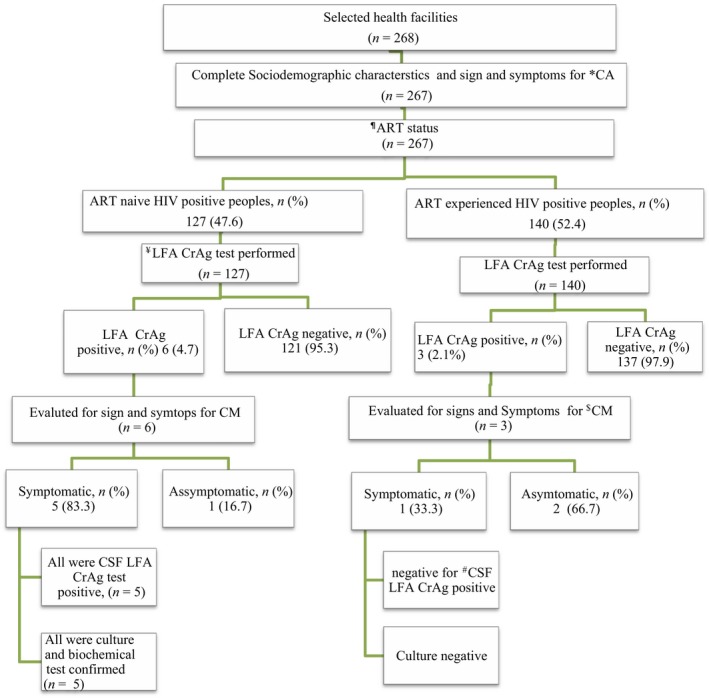
Study participant recruitment flowchart: ^¶^ART: antiretroviral therapy; *CA: cryptococcal antigenemia; ^$^CM: cryptococcal meningitis; ^#^CSF: cerebrospinal fluid; ^¥^LFA CrAg: lateral flow assay cryptococcal antigen

Out of 280 respondents, a complete response (with socio‐demographic, clinical, and laboratory data) was obtained for only 267 study participants. Of these 267 study participants, about half 51% (*n* = 137) were females and 222 (83.1%) were urban residents. The mean (±*SD*) age was 38 (±10) years. Majority (86.5%) were on outpatient management, and 140 (52.4%) were on ART. At least one or more sign and symptoms were reported from all the study participants. Therefore, from all study participants, 33% (*n* = 87) had headache, 31% (*n* = 83) had cough, 28.8% (*n* = 77) had fever, 27.3% (*n* = 73) had nausea, 24.3% (*n* = 65) had vomiting, 20.6% (*n* = 55) had visual changes, and 11.6% (*n* = 31) had neck stiffness irrespective of CrAg status **(**Table 1 **,** Figure 1 **)**.

**Table 1:** Cryptococcal antigenemia among ART‐naïve and ART‐initiated peoples (*n* = 267)

	ART naïve (*n* = 127)					On ART (*n* = 140)		
Variables	CrAg+:*n*(%)		CrAg−: *n*(%)		Total: *n*(%)	CrAg+:*n*(%)	CrAg−: *n*(%)	Total:*n*(%)
Gender								
Female		1 (1.5)		67 (98.5)	68 (53.5)	0 (0.00)	69 (100.0)	69 (49.3)
Male		5 (8.5)		54 (91.5)	59 (46.5)	3 (4.2)	68 (95.8)	71 (50.7)
Age (Years)								
18–27		1 (4.8)		20 (95.2)	21 (16.5)	0 (0.0)	11 (100.0)	11 (7.6)
28–37		3 (5.5)		52 (94.5)	55 (43.3)	1 (2.3)	43 (97.7)	44 (31.4)
38–47		2 (4.9)		39 (95.1)	41 (32.3)	2 (4.1)	47 (95.9)	49 (35)
48–57		0 (0.0)		7 (100.0)	7 (5.5)	0 (0.0)	22 (100.0)	22 (15.7)
58–67		0 (0.0)		3 (100.0)	3 (2.4)	0 (0.0)	12 (100.0)	12 (8.6)
68–75		—		—	—	0 (0.0)	2 (100.0)	2 (1.4)
Residence								
Urban		3 (2.8)		103 (97.2)	106 (83.5)	1 (0.9)	115 (99.1)	116 (82.6)
Rural		3 (14.3)		18 (85.7)	21 (16.5)	2 (8.3)	22 (91.7)	24 (17.1)
Patients								
Out patients		1 (1.0)		102 (99.0)	103 (81.1)	0 (0.0)	128 (100.0)	128 (91.4)
Inpatients		5 (20.8)		19 (79.2)	24 (18.9)	3 (25.0)	9 (75.0)	12 (8.6)
CD4 count (cells/mm^3^)								
≤50		4 (12.9)		27 (87.1)	31 (50.8)	2 (6.7)	28 (93.3)	30 (42.5)
51–100		1 (4.0)		24 (96.0)	25 (53.2)	1 (4.5)	21 (95.5)	22 (46.8)
101–150		1 (4.0)		24 (96.0)	25 (50.0)	0 (0.0)	25 (100.0)	25 (50.0)
>150		0 (0.0)		46 (100.0)	46 (42.2)	0 (0.0)	63 (100.0)	63 (57.8)
WHO stage								
Stage I		0 (0.0)		31 (100.0)	31 (24.4)	0 (0.0)	102 (100.0)	102 (72.9)
Stage II		0 (0.0)		31 (100.0)	31 (24.4)	0 (0.0)	12 (100.0)	12 (8.6)
Stage III		0 (0.0)		40 (100.0)	40 (31.5)	1 (6.7)	14 (93.3)	15 (10.7)
Stage IV		6 (24.0)		19 (76.8)	25 (19.7)	2 (18.2)	9 (81.8)	11 (7.9)
Sign and symptoms								
Headache		4 (8.5)		43 (91.5)	47 (54.0)	2 (6.7)	28 (93.3)	30 (46.0)
Cough		4 (6.7)		56 (93.3)	60 (72.3)	3 (13.0)	20 (87.0)	23 (27.7)
Fever		2 (3.5)		55 (96.5)	57 (74.0)	2 (10.0)	18 (90.0)	20 (26.0)
Nausea		5 (13.2)		33 (86.8)	38 (52.1)	2 (5.1)	33 (94.9)	35 (47.9)
Vomiting		6 (15.4)		33 (84.6)	39 (60.0)	1 (3.8)	25 (96.2)	26 (40.0)
Visual change		3 (7.5)		37 (92.5)	40 (72.7)	1 (6.7)	14 (93.3)	15 (27.3)
Neck stiffness		3 (13.6)		19 (86.4)	22 (70.9)	0 (0)	9 (100)	9 (29.1)

Note

CrAg+: positive for cryptococcal antigenemia; CrAg−: negative for cryptococcal antigenemia.

### 3.2 Prevalence of cryptococcal antigenemia

The prevalence of cryptococcal antigenemia was 9 (3.4%). All the study participants, except one (CD4 = 120 cells/mm^3^), had CD4 count less than 100 cells/mm^3^. The cryptococcal antigenemia‐positive peoples had one or more clinical sign and symptoms, of which, 6 (6.9%) had a headache, 4 (5.2%) had a fever, and 3 (9.7%) had neck stiffness. Six of the CrAg positives were ART naive, and 3 were ART experienced. From the total study participants with a CD4 count less than or equal to 50** **cells/mm^3^, 6 (9.8%) were with cryptococcal antigenemia. Considering their ART status, among the ART‐naïve groups from those with a CD4 count of less than or equal to 50 cells/mm^3^, 4 (12.9) were positive for *Cryptococcus*. However, only one study participant with a CD4 count of 120 cells/mm^3^ was positive to *Cryptococcus*. Eight of the nine CrAg positive were on WHO stage IV of HIV/AIDS (Table 1).

### 3.3 Cryptococcal meningitis among cryptococcal antigenemia‐positive peoples

Cryptococcal antigen‐positive peoples were examined for signs and symptoms of meningitis. From 9 peoples with antigenemia, 5 from ART naïve and 1 from ART‐experienced groups had one or more signs and symptoms indicative for cryptococcal meningitis. Lumbar puncture was performed on those 6 study participant, of which, only 5 were positive for CSF LFA CrAg test. All CrAg positives were confirmed by culture and biochemical test (Figure 1 **)**.

### 3.4 Factors associated with cryptococcal infection

Among risk factors, being male (*p* = 0.017), living in rural areas (*p* = 0.008), and being hospitalized (*p* = 0.001) were statistically associated with cryptococcal antigenemia (Table 2).

**Table 2:** Associated risk factors with cryptococcal antigenemia (*n* = 267)

Variables	CrAg+ (*n* = 9): *n* (%)		CrAg− (*n* = 258): *n* (%)	*p*‐value*
Gender				
Female	1 (0.7)		136 (99.8)	1
Male	8 (6.2)		122 (93.8)	0.017*
Age (Years)				
18–27		1 (3.1)	31 (96.9)	
28–37		4 (4.0)	95 (96.0)	
38–47		4 (4.4)	86 (95.6)	NA
48–57		0 (0.0)	29 (100.0)	
58–67		0 (0.0)	15 (100.0)	
68–75		0 (0.0)	2 (100.0)	
Residence				
Urban		4 (1.8)	218 (98.2)	1
Rural		5 (11.1)	40 (88.9)	0.008*
Patients				
Out patients		1 (0.4)	230 (99.6)	1
Inpatient		8 (22.2)	28 (77.8)	0.001*
CD4 count(cells/µl)				
≥100		1 (0.6)	159 (99.4)	1
<100		8 (7.5)	99 (92.5)	0.003*
ART status				
ART naive		6 (4.7)	121 (95.3)	1
On ART		3 (2.1)	137 (97.9)	0.317

Note

CrAg+: positive for cryptococcal antigenemia; CrAg−: negative for cryptococcal antigenemia.

a Fisher's exact test.

## 4 DISCUSSION

HIV‐infected peoples are at risk for different opportunistic infections, including fungal infections. Late presentations to care of HIV‐infected peoples will put them at risk for the development of the cryptococcal disease. In resource constraining settings, continuous surveillance of the cryptococcal infection in HIV‐infected peoples and associated factors is important to support national program related.

The prevalence of cryptococcal antigenemia in the current study is relatively higher than the prevalence reported from Adama (1.6%) (Reepalu et al., 2015) and much lower than the findings from Addis Ababa (8.4%) (Alemu et al., 2013) and Adama (10.2%) (Beyene et al., 2013). The difference in the prevalence rate might be due to improvements in a time of ART initiation for HIV‐infected peoples, which might reduce the incidence and high mortality associated with cryptococcal meningitis and other opportunistic infections (Alemu et al., 2013). However, in contrast to these, in a recently conducted study on HIV‐initiated peoples with a CD4 count of less than 150 cells/mm^3^ showed a prevalence of 6.2% (Beyene et al., 2017). The difference could be due to a large number of study participants with lower CD4 count are included (Beyene et al., 2017).

We compared our findings with other studies conducted outside Ethiopia. The findings from this study were similar to recent prevalence reports from Namibia (3.3%) (Sawadogo et al., 2016) and Tanzania (3.7%) (Letang et al., 2015) and slightly lower than the prevalence reported in South Africa (4.3%) (Longley et al., 2016). However, lower than the prevalence reported in two studies from Nigeria (12.7% and 8.9%) (Oladele et al., 2016; Osazuwa, Dirisu, Okuonghae, & Ugbebor, 2012) and two more from Tanzania (7.1% and 5.1%) (Magambo et al., 2014; Wajanga et al., 2011). We also compared our finding with studies in other developing countries like Indonesia (7.1%) (Ganiem et al., 2014), Vietnam (4%) (Smith et al., 2013), and Thailand (9.2%) (Pongsai, Atamasirikul, & Sungkanuparph, 2010). The reasons for the wide difference in the prevalence reports even within the same countries might be due to the difference in sample size, participant selection, study design, and seasonal variations on which the studies were conducted (Randhawa et al., 2011).

Considering cryptococcal meningitis (CM), in this study, high CM rates among ART‐naïve study participants were found. This is similar to other studies in Sub‐Saharan Africa. For example, in South Africa, CM was found 4/10 antigenemia‐positive cases (Longley et al., 2016), in Cameroon 41/146 of patients who had clinical signs of meningitis (Ngouana et al., 2015), and in Tanzania 15/17 cryptococcal antigenemia‐positive hospitalized patients (Wajanga et al., 2011). But we are unable to discuss further the findings from a statistical point of view due to the few numbers of study participants with CM.

However, in this study males, residing in rural areas, CD4 < 100 cells/mm^3^, and being hospitalized were statistically associated with cryptococcal antigenemia. Although cryptococcal infections occur in both sexes, a statistically significant difference in gender was also observed in other studies; this is likely due to poor health‐seeking behavior of men to present later to care (Alemu et al., 2013; Assogoba et al., 2015; Ngouana et al., 2015) and the interaction *Cryptococcus* with testosterone, which results in increased capsular polysaccharide release and *Cryptococcus*‐mediated macrophage death (McClelland et al., 2013). In addition to this, rural residents were at risk for cryptococcal infection. This might be supported by genotyping studies that dealt with the ubiquitous nature of *Cryptococcus* species providing strong evidence for additional origins of exposure like different species of plants, soil, and house dust (Chen et al., 2015; Cogliati, 2013). From the sign and symptoms, nausea and vomiting were observed in most of the participants tested positive in contrary to other studies (Hashimoto e Souza et al., 2013; Jarvis, Meintjes, Williams, Rebe, & Harrison, 2010; Makadzange & McHugh, 2014). In addition to this, those that were hospitalized for different reasons were at a significant risk of cryptococcal infections (Kaplan et al., 2015). In this study, higher prevalence of CrAg was observed in peoples with advanced immune‐suppression, which is supported by other similar studies, and WHO recommendations (Alemu et al., 2013; Ganiem et al., 2014; Osazuwa et al., 2012; Pongsai et al., 2010; Randhawa et al., 2011; Sawadogo et al., 2016; Smith et al., 2013; World Health Organization, 2011). Moreover, though a significant association was not found on their ART status, higher prevalence of *Cryptococcus* was observed in those that were ART naïve. Preventing new HIV infections, early HIV diagnosis, implementing early linkage to care, and ensuring timely initiation of ART with strict adherence would prevent incident cryptococcosis including Cryptococcal antigen screening and preemptive therapy with fluconazole (Asfaw et al., 2015; Smitson et al., 2014). Therefore, resource‐constrained settings, including Ethiopia, should work for improving their national program for the early ART initiation and promoting a cost‐effective preventive strategy to reduce the incidence and high mortality associated with cryptococcal meningitis in peoples with low CD4 count (Jarvis et al., 2013).

The limitations of this study are as follows: we were unable to see the independent effect of variables on cryptococcal antigenemia. It was also difficult to perform statistical analysis for Cryptococcal meningitis due to small sample size.

## 5 CONCLUSION

This study revealed that cryptococcal infection poses a substantial risk of HIV/AIDS‐infected peoples despite the widespread use of HAART. High prevalence of cryptococcal infection in ART‐naïve peoples was observed. Moreover, being male, living in rural areas, low CD4 count, and being hospitalized were statistically associated with cryptococcal infection. This provides relevant data for CrAg screening interventions in patients with low CD4 cell counts.

## CONFLICT OF INTEREST

The authors have no competing interests to declare.

## AUTHOR CONTRIBUTION

HK participated in the conception of the title and study design, data collection, data analysis, data entry, and writing the manuscript. NS participated in the study during design and analysis. KH participated in data cleaning. AM was involved in the design and study monitoring. All authors read and approved the final manuscript.

## Data Availability

The data will be available on request from the corresponding authors following permission from Research Ethics Review Committee of the College of Health Sciences, Mekelle University.
